# Recovery of methamphetamine associated cardiomyopathy predicted by late gadolinium enhanced cardiovascular magnetic resonance

**DOI:** 10.1186/1532-429X-11-46

**Published:** 2009-11-11

**Authors:** Javier E Lopez, Khung Yeo, Gary Caputo, Michael Buonocore, Saul Schaefer

**Affiliations:** 1 Department of Internal Medicine, Division of Cardiovascular Medicine, One Shields Avenue, Davis CA 95618, USA; 2 Department of Radiology, University of California Davis Medical Center, 2315 Stockton Boulevard, Sacramento, CA 95817, USA

## Abstract

Methamphetamine is known to cause a cardiomyopathy which may be reversible with appropriate medical therapy and cessation of use. Late gadolinium enhancement cardiovascular magnetic resonance (CMR) has been shown to identify fibrosis in ischemic and non-ischemic cardiomyopathies. We present a case of severe methamphetamine-associated cardiomyopathy in which cardiac function recovered after 6 months. Evaluation by CMR using late gadolinium enhancement was notable for an absence of enhancement, suggesting an absence of irreversible myocyte injury and a good prognosis. CMR may be useful to predict recovery in toxin-associated non-ischemic cardiomyopathies.

## Background

Methamphetamine is a synthetic amine commonly used as a recreational drug because of its stimulant effects. Its use has increased nationwide [1] and recent reports suggest that methamphetamine use is present in at least 5% of all patients presenting to the emergency room with heart failure [2] and 40% of patients under the age of 45 admitted to the hospital with cardiomyopathy [3]. Chronic use has also been associated with the development of chronic coronary disease [4,5] as well as cardiomyopathy [3,6].

Recovery of left ventricular dysfunction in patients with methamphetamine-induced cardiomyopathy has been described [7-9]. However, since the effects of methamphetamine can include myocyte hypertrophy [10] and fibrosis [11,12], both relatively irreversible processes, it is likely that some patients will not recover left ventricular function with either appropriate medical therapy or abstinence from methamphetamine.

Cardiovascular magnetic resonance (CMR) with late gadolinium enhancement (LGE) has been show to identify myocardial fibrosis in ischemic and non-ischemic cardiomyopathies [13,14] and provide prognostic information about cardiac recovery in these disease processes [15]. LGE has not, to our knowledge, been used to evaluate fibrosis and predict recovery in methamphetamine-associated cardiomyopathy.

We herein report a case of recovery of left ventricular function in a patient with methamphetamine-associated cardiomyopathy demonstrated using LGE.

## Case report

**Figure 1:**
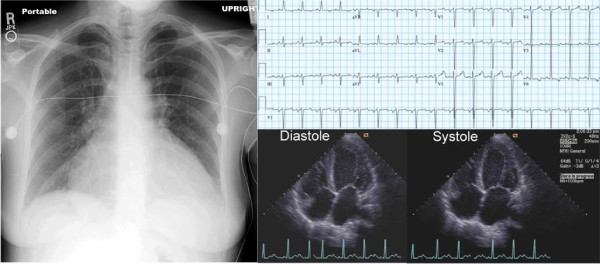
**Chest X-ray, electrocardiogram, and 2-D echocardiograms of the patient on initial evaluation**. These examinations demonstrated cardiomegaly, left ventricular hypertrophy with ST-T wave changes, and decreased left ventricular systolic function.

A 44 year old woman presented to the emergency department with 3 days of peripheral edema, paroxysmal nocturnal dyspnea, and dyspnea on exertion. She denied chest pain, fever, rashes and/or recent viral illnesses. She had no recent exposure to ill contacts or foreign travel. Her review of systems was otherwise unremarkable. Her past medical history consisted of pregnancy related hypertension 4 years prior that required no therapy after delivery. Her social history consisted of 15 years of inhaled methamphetamine and tobacco use. She denied alcohol or other street drug use. She was taking no medications at time of presentation. Her surgical history consisted of one normal child delivery 4 years prior, a tubal ligation and tonsillectomy. Physical examination revealed a blood pressure of 159/109 mmHg, pulse of 109 bpm, and a temperature of 36.3°C. Her arterial O_2 _saturation was 99% on room air. Cardiac examination revealed a JVP of 12 cm and a 3/6 holosystolic murmur best heard at the apex without respiratory variation. She had no organomegaly or ascites and her extremities had 2+ pitting edema. Her laboratory data demonstrated a sodium of 138 mEq/L, creatinine of 1.1, and serum troponin of 0.11 (ng/ml). A chest X-ray showed cardiomegaly, an electrocardiogram showed left ventricular hypertrophy and ST-T wave abnormalities. Two-dimensional echocardiography showed reduced systolic function (Figure 1).

After informed consent, she underwent LGE using standard techniques [16]. Briefly, images were acquired on a Siemens 3T Trio MR system using a 4 element cardiac array coil. After localization scans, CINE sequences were run in three planes for assessment of wall motion and ejection fraction. The contrast agent (gadolinium-DTPA, 0.2 ml/Kg) was injected and a set of inversion recovery (IR) gradient recalled echo (GRE) sequences was run with different TI values starting 5 min after injection. Following determination of the optimal TI value, IR-GRE "delayed enhancement" images were acquired in the short axis, vertical long axis and horizontal long axis orientations 10-20 minutes after the injection of the contrast agents. One to eight slice locations were acquired for each orientation. Images were analyzed in duplicate using a Leonardo workstation (Siemens Medical Solutions, Erlangen, Germany). Ejection fraction and left ventricular mass were calculated by computer-assisted endocardial border definition of end-diastolic and end-systolic frames. The presence of gadolinium enhancement was defined as pixel intensity in the myocardium > 3× background [16].

**Figure 2:**
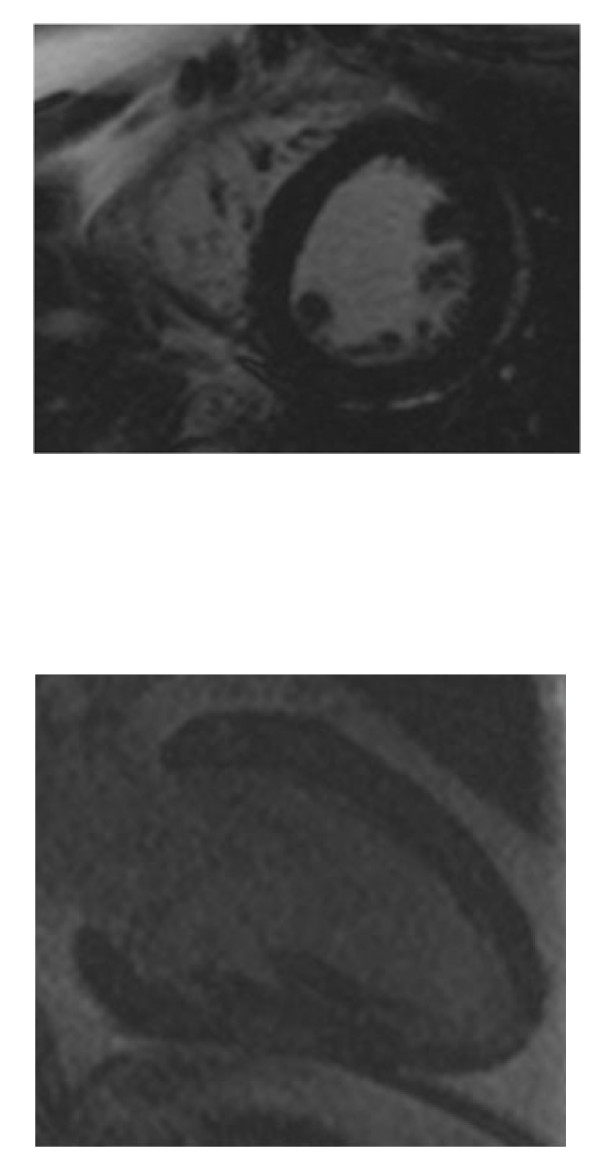
**Late gadolinium enhancement short-axis and vertical long axis images**. There was no myocardial enhancement (black).

Echocardiography and CMR both showed a reduced ejection fraction, calculated at 37% using quantitative analysis of GRE images. There was no LGE (Figure 2). She was placed on medical therapy including a beta-blockers and an angiotensin converting enzyme inhibitor and was advised to abstain from methamphetamine use.

**Figure 3:**
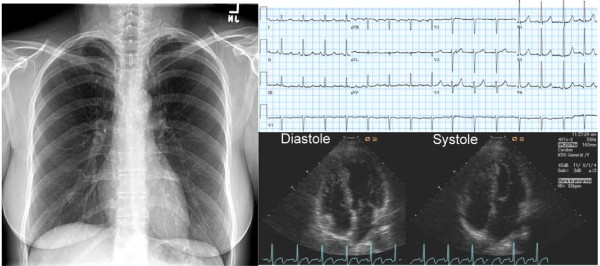
**Chest X-ray, electrocardiogram, and 2D echocardiograms of the patient after 6 months of therapy**. The cardiac silhouette is now normal, the ST-T changes on ECG have resolved, and the 2-D echocardiogram shows normal systolic function.

Following 6 months of medical therapy and decreased use of methamphetamine, she again was evaluated for signs and symptoms of heart failure. At this time, she had improved clinically to functional class I. Echocardiography showed a normal ejection fraction and quantitative CMR showed an ejection fraction of 64% (Figure 3). Left ventricular mass changed from 234 to 185 grams.

## Discussion

This patient illustrates the potential use of LGE as a tool to predict whether patients with methamphetamine-associated cardiomyopathy can recover left ventricular function with appropriate medical therapy. In this instance, the absence of gadolinium enhancement was consistent with an absence of macroscopic regions of fibrosis and hence, no irreversible myocardial injury.

Methamphetamine is a sympathomimetic agent that mediates its cardiovascular effects through excessive release of norepinephrine and blockade of reuptake at the sympathetic synaptic receptors [17]. This sympathetic stimulation can cause either acute ventricular dysfunction, such as seen in Takotsubo syndrome [18], or chronic left ventricular dysfunction [9]. Components of left ventricular dysfunction include both reversible events such as myocardial stunning [19] and irreversible changes including myocyte loss and replacement fibrosis [11]. Animal studies with 12 week exposure to methamphetamine have not only shown cellular changes such as atrophy, hypertrophy, patchy cellular infiltration, and fibrosis, but have also demonstrated gradual recovery starting 3 weeks after cessation of exposure [11]. In patients, there are isolated case reports suggesting that methamphetamine associated cardiomyopathy is reversible with discontinuation of abuse [8,9].

While the extent of fibrosis defined by LGE predicts recovery of left ventricular function in patients with coronary artery disease and myocardial infarction [16], there are insufficient data regarding functional recovery in patients with non-ischemic cardiomyopathy [20]. However, the extent of fibrosis in patients with non-ischemic cardiomyopathy, as identified by LGE, predicts event free survival [15].

## Conclusion

In the specific case of this patient with methamphetamine-associated cardiomyopathy, the LGE study did not demonstrate any enhancement, consistent with an absence of significant fibrosis. Left ventricular function recovered with 6 months of medical therapy and decreased drug abuse. While it is unknown whether, in a larger cohort of patients, the absence or presence of CMR identified fibrosis would predict recovery, the benign LGE findings in this patient likely portended a favorable outcome.

## Competing interests

The authors declare that they have no competing interests.

## Authors' contributions

JEL and SS analyzed the data and wrote the manuscript. KK and SS conceived of the study, and participated in its design and coordination. GC participated in the acquisition of the data. MB participated in the acquisition and analysis of the data. All authors read and approved the final manuscript.

## Consent

Written informed consent was obtained from the patient for publication of this case report and accompanying images. A copy of the written consent is available for review by the Editor-in-Chief of this journal.
